# Ecological Water Requirement in Upper and Middle Reaches of the Yellow River Based on Flow Components and Hydraulic Index

**DOI:** 10.3390/ijerph182010956

**Published:** 2021-10-18

**Authors:** Shibao Lu, Wenting Cai, Wei Shao, Farhad Taghizadeh-Hesary, Muhammad Faisal, Hongbo Zhang, Yangang Xue

**Affiliations:** 1 School of Public Administration, Zhejiang University of Finance and Economics, Hangzhou 310018, China; lushibao@zufe.edu.cn; 2 School of Political Science and Public Administration, Neijiang Normal University, Neijiang 641000, China; 3 China Urban Construction Design & Research Institute Co., Ltd., Beijing 100120, China; caiwenting@zufe.edu.cn; 4 China Research Institute of Regulation and Public Policy, Zhejiang University of Finance and Economics, Hangzhou 310018, China; 5 School of Economics, Zhejiang University of Finance and Economics, Hangzhou 310018, China; 6 Social Science Research Institute, Tokai University, Hiratsuka-shi 259-1292, Kanagawa-ken, Japan; 7 College of Economics and Management, Huazhong Agricultural University, Wuhan 430070, China; faisalgurmani@gmail.com; 8 School of Environmental Science and Engineering, Chang’an University, Xi’an 710054, China; zhanghb@chd.edu.cn; 9 School of Electrical Engineering, Lanzhou Institute of Technology, Lanzhou 730050, China; xyg3024_cn@163.com

**Keywords:** Yellow River, ecological environment, fish survival, lowest flow, suitable flow

## Abstract

Deterioration of the ecological environment in the upper and middle reaches of the Yellow River in China substantially impacts the growth and development of aquatic organisms in the drainage basin. This paper builds a conceptual model by applying flow components and fish ecological requirements relation with a relevant object of main fish in the upper and middle reaches of the Yellow River. The paper utilized the flow restoration method by employing the River2D model (two-dimensional model of river hydrodynamics and fish habitat), and a one-dimensional hydrodynamics HEC-RAS (hydrologic engineering center’s-river analysis system). The calculation result showed that the runoff condition required for *Silurus lanzhouensis* survival is that the monthly lowest flow in a year is 150 m^3^·s^−1^, and the lowest flow for suitable flow from April to October is 150 m^3^·s^−1^, and 300 m^3^·s^−1^ from November to March. The research result is closer to the actual condition and has more outstanding operability. Meanwhile, the results proposed the coupling method of ecological water requirement for the mainstream of the Yellow River. Moreover, the results portrayed the ecological flow process according to the upper envelope of minimum and maximum ecological water requirements of each fracture surface. It is regarded that the ecological flow process is deemed as the initial value of the reservoir regulation model.

## 1. Introduction

With the rapid development of industrial civilization, humans significantly impact, occupy, and control water resources on a specific spatial and temporal scale. Water resources gradually lose their ecological functions and lead to severe ecological environmental problems [1,2,3,4,5]. Therefore, the water resource should be detected and regulated scientifically as the ecological system needs to be protected and restored, i.e., the ecological research of the river flow should be foremost conducted. The river flow here refers to the water volume, which is the central part of the ecological structure integrity and biological diversity [6,7,8,9,10]. According to the literature, 44 countries in the world calculated the ecological flow of the river with different methods, and results demonstrated the volume exceeds more than 200 m^3^·s^−1^ [11,12,13,14]. These methods can be divided into four categories: hydrological method, hydraulic method, habitat method, and global analytical method. The habitat method is a quantitative method based on the biology principle, which integrated the relationship between preferential habitat characteristics of biology and river hydrologic characteristics, being considered the most reliable evaluation method at present. The most common technique in habitat methods is the instream flow incremental methodology (IFIM), and this technique combines a lot of hydrographic measurement data with the specific aquatic organism [15,16,17]. The IFIM method is not widely applied in America, but it is often used in more than 20 countries like France, Germany, Japan, the Czech Republic, and England [15]. At present, mostly large and middle river research takes fish as the indicating organism. Nevertheless, in a mountain stream with a lower place, the diversity of sizeable benthic animals is higher than that of fish, which directly and closely relates to the environmental factor of habitat. This research can be applied in the USA, Canada, New Zealand, and Australia [18,19,20,21,22].

The main object of ecological construction is to reduce soil and water loss [23]. Primarily, it increases the vegetation coverage by directly utilizing precipitation enhancement to reduce runoff [24], which is the exceptional ecological efficiency in the Yellow River basin. Part of river runoff is transferred to slope surface ecological water utilization. One crucial consequence caused by insufficient runoff is severe riverbed deposition and river course shrinkage [25]. Levee at both sides in the lower reaches of the Yellow River has deposited about 10 billion tons, and the riverbed is raised 2–4 m. Since the 1990s, the annual average deposit in the lower reaches of the Yellow River is 220 million tons; a long-term small flow process results in severe primary channel deposition in the lower reaches of the Yellow River. Based on statistical analysis, primary channel deposition in the lower reaches of the Yellow River occupies 90% of the deposition volume of the whole fracture surface (such as Huayuankou to Gao Village). Serious deposition of river channel causes severe shrinkage of the cross-section of the river, with the result that flow water level raises, bank full discharge reduces dramatically. The main channel in the lower reaches of the Yellow River has shrunk, and the flood-carrying capacity of the channel has dropped, which brings serious difficulties to flood protection [26].

River shrinkage results in the degradation of the water ecosystem and a reduction in biological diversity [27]. The sediment concentration in the water of the Yellow River is substantial; sunlight is difficult to transmit, which limits the growth and reproduction of light plants and animals [22]. Compared with other rivers, the Yellow River’s biological diversity is not rich, including carp, *Cololabis caira Brevoort* [28], and *Coreius heterodon* [29]. In the past 10 years, the river has been badly polluted, some aquatic organisms are in imminent danger, the business yield of natural fish in the Yellow River is virtually zero, and migratory fish are nearly extinct [26]. The gradual reduction in water volume in the river course has also impacted the water quality; simultaneously, sewage has not been effectively controlled, which is directly discharged into the water [27]. The water quality pollution has been intensified. A reduction in drying of the water body surface such as rivers and lakes can neither meet the ecological demands nor produce the dilution and purification effect to the pollutants. The pollution of the surface water body, underground water body, and even offshore area has increased [19].

Compared with other rivers in China, research on the ecological requirements of the Yellow River are significant in both theory and practice. Ecological water requirement research is an exploration process with continuous improvement. With a deepened understanding of the river ecological hydrology relation, this paper determines hydraulic parameters of different flow components by referring to the hydrologic condition of the drainage basin. The hydraulic model was used to calculate the ecological water requirement of fish in the upper and middle reaches of the Yellow River, which accords with the actual condition of fish survival in the area.

## 2. Materials and Methodologies

### 2.1. Study Area

**Figure 1:**
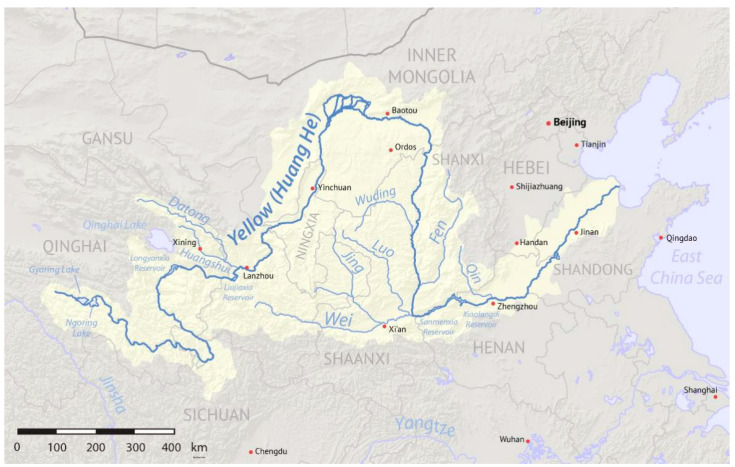
The upper and middle Yellow River drainage system diagram. Source: China Water Resources Yearbook, 2018.

The Yellow River basin has a vast territory, which crosses three major geographical terrains in China (Figure 1). The full length of the river course for the mainstream is 5464 km, and the annual mean natural runoff volume of the full river for many years is 58 billion m^3^·s^−1^, which only occupies 2% of river runoff volume in China [26].

The Yellow River source area maintains the essential flowing water of the Yellow River’s life by taking a 49% runoff volume of the overall drainage basin. With global climate warming, the annual average temperature in the Yellow River area has the same trend. The snow line rises; the ground temperature rises; the elevation of the lower bound for frozen earth rises, which brings a series of ecological problems. Water and soil loss are the traditional ecological problem in the Yellow River, and this kind of problem is most apparent on the Loess plateau. The soil layer in the Loess plateau is profound, and the soil property is loosening. The terrain is crushed; the rainstorm is frequent, water and soil loss are severe, which is the primary source of sediment of the Yellow River and has caused the heterogeneous situation of the flow and sediment. The area of the upper and middle reaches accounts for 97% of the total area. The riverbed stretching for hundreds of kilometers in the lower reaches of the Yellow River is higher than the ground on both banks. A drainage area of the riverbed only accounts for 3% of the total area [26].

### 2.2. Methodologies

**Figure 2:**
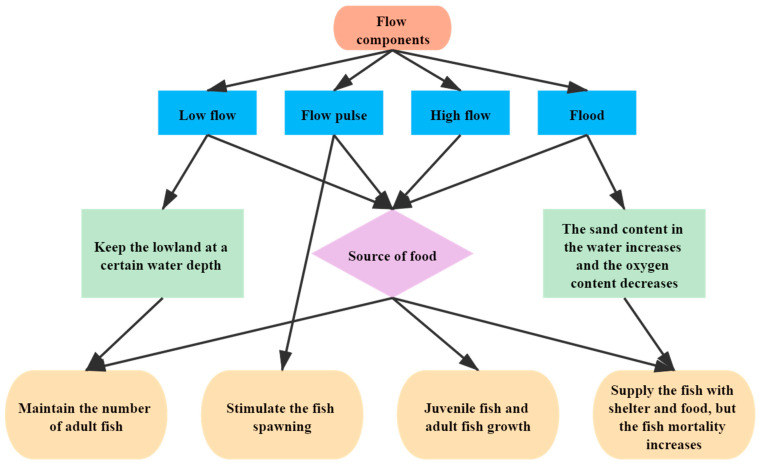
Conceptual model of the relationship between *Silurus lanzhouensis*, *R. nasutus*, and flow components; Source: Authors’ depiction.

According to QingYu [30], the essential ecological habit of *Silurus lanzhouensis and Rhinogobio nasutus* in the upper and middle reaches of the Yellow River, the conceptual model is shown in Figure 2. The Wang [26] model indicates the relationship between *Silurus lanzhouensis* and *S. lanzhouensis, R. nasutus* and the component of the flow. Due to the lack of a specific hydraulic index of different flow components for the above two fish species, it is hard to give a specific quantity value of each flow component by the hydraulic model. However, research has shown that two flow conditions should be met [30]. The first is basic water depth condition for fish survival in non-flood season; the second is the water flow condition required for spawning site in the spawning period. From the requirement of *Silurus lanzhouensis* spawning, *Silurus lanzhouensis* spawns in shallow water grass on the bank with sticky eggs. Therefore, a larger flow submerged shallow area is required [26]. Although flood is not essential for fish survival, the flood with appropriate frequency is beneficial to fish feeding and expands the chance for survival [9].

#### 2.2.1. Comparing River2D with IFIM 

The River2D model is a two-dimensional average depth model for river hydrodynamics and fish habitat. It is an application software for hydraulic and fish habitat simulation. River2D can study the river’s local velocity and depth distribution in detail and determine the relationship between the flow and the available habitat of fish. It has been highly applied in the process of bridge design, river regulation, pollutant migration, and fish habitat evaluation. The River2D model is generally a two-dimensional average depth model of river hydrodynamics and fish habitat [31].

The instream flow incremental methodology (IFIM) is a decision support system for river planning, protection, and management. It consists of a series of professional models and various methods such as hydrodynamics, water quality, hydrology, and ecology [26]. The hydraulic model and the biological information model are combined to establish the quantitative relationship between the flow and the suitable habitat of fish, and then the time series of the habitat is determined by the hydrological model to provide a scientific basis for water resources planning. Fish is selected as an indicator species by the IFIM method. On the one hand, fish is one the most sensitive species to environmental changes because it is at the top of the food chain of aquatic communities [32]. As a top community, fish play an important role in the existence and abundance of other species. On the other hand, the impact of flow variability and habitat management on fish habitats is evaluated by establishing the relationship between the quantity and quality of available habitats and the flow.

The IFIM method is a theoretical system framework that provides a basis for water resource planning by simulating the relationship between species’ available habitat and flow. It does not produce a specific flow value in the river by itself, but it is determined through negotiations by all parties of the water user. When the IFIM method is used to study the water demand of the river’s ecological environment, there must be sufficient data on the relationship between the flow and time. In the IFIM method, a variety of models can be used, and hydraulic models can be selected from one-dimensional to three-dimensional according to research needs [15,33]. 

#### 2.2.2. The Relevant Object of Main Fish in the Upper and Middle Reaches of the Yellow River and Flow Components

The annual distribution of ecological water demand is not balanced. It is highest for water requirement from April to June. The second place is from July to October, and the minimum demand is from January to March and November to December. This reflects that fish have different preferences to flow from the spawning period (April to June), the larval stage (July to October), and the growth period (January to March and November to December). In order to simulate the spawn, the flow rate in the spawning period is more extensive, which is typically the upper limit for fish-prefer flow rate; the preferential flow rate in the larval stage is moderate, the preferential flow rate in the growth period is the typically lower limit of the preferential flow rate [34,35,36]. Table 1 shows the ecological object, corresponding to the flow component, hydraulic index, and time distribution for *Silurus lanzhouensis*. Because of a lack of ecological habit data for *Rhinogobio nasutus*, the related research will not be shown. Ecological flow is calculated by the ecological habit of *Silurus lanzhouensis* and the Yellow River carp in middle reach.

#### 2.2.3. Determine Hydraulic Parameters of Different Flow Components by Using the Hydraulics Model

The habitat area is obtained according to the fish to the flow rate and water depth by applying the above two-dimensional hydraulic model River2D to calculate ecological flow, which is a simple method. Due to missing terrain data in upper and middle reaches, the HEC-RAS (hydrologic engineering center’s-river analysis system) one-dimensional hydraulic model was developed by the United States Army Corps of Engineers, which is required for the ecological water requirement of fish in upper and lower reaches [21,32,37,38,39,40]. The model can calculate hydraulic parameters of different fracture surfaces in different flow conditions. These parameters include maximum water depth, average depth, cross-section area, wetted perimeter, average flow rate, and so on (see Figure 2). The model can be downloaded to use for free at http://www.hec.usace.army.mil/SOFTWARE/hec-ras/ (accessed on 8 October 2021).

## 3. Results

### 3.1. Corresponding Hydraulics Parameters Calculation with Different Environment Components

**Figure 3:**
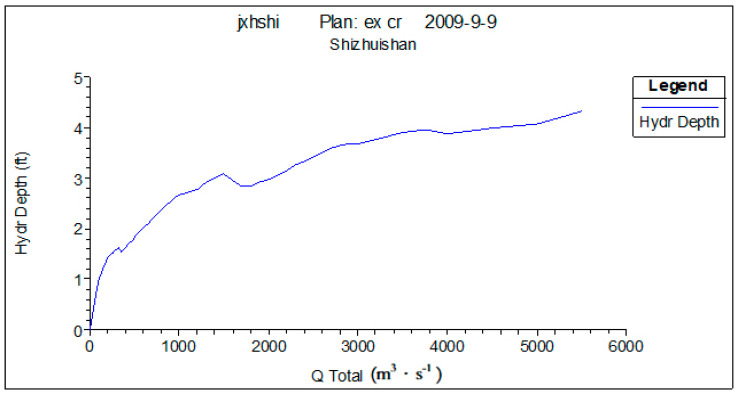
Water depth change at Shizuishan cross under different flows. Source: China Water Resources Yearbook, 2018.

**Figure 4:**
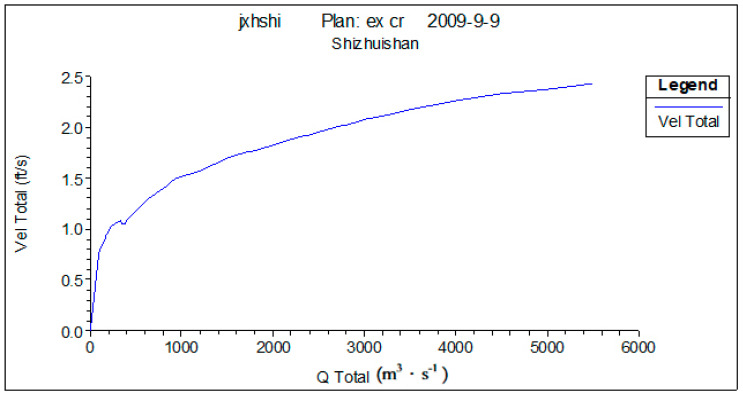
Flow velocity change at Shizuishan cross under different flows. Source: China Water Resources Yearbook, 2018.

**Figure 5:**
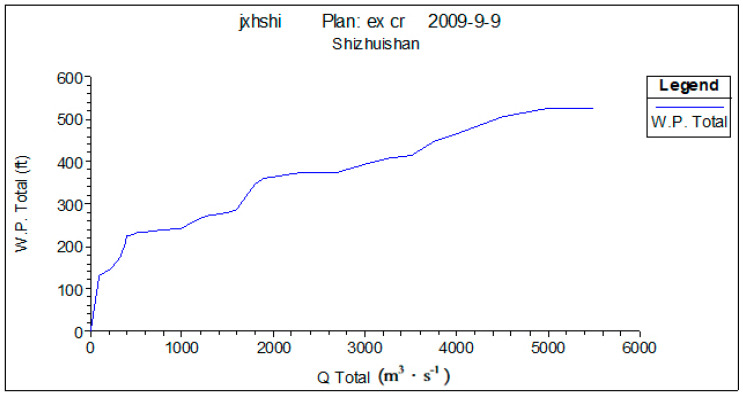
Change at Shizuishan cross under different flows. Source: China Water Resources Yearbook, 2018.

Four fracture surfaces are selected in the lower reaches of the Yellow River to describe the hydraulic characteristics of the Yellow River’s lower reaches. These four fracture surfaces are Shizuishan, Toudaoguai, Longmen, and Tongguan. Figure 3, Figure 4 and Figure 5 show the water depth, flow rate, and wetted perimeter change conditions in different flows of the Shizuishan fracture surface.

In these figures, we can see that when the low flow is about 150 m^3^·s^−1^ from November to March, the water depth and flow rate can meet the survival necessity of *Silurus lanzhouensis*. Therefore, research suggests that the low limit flow satisfying for *Silurus lanzhouensis* in Shizuishan is 150 m^3^·s^−1^.

In terms of habitat conditions required for fish from April to June, when the flow is greater than 400 m^3^·s^−1^, the wetted perimeter increases slowly, which means 400 m^3^·s^−1^ is the inflection point of wetted perimeter change, i.e., when the flow is greater than 400 m^3^·s^−1^, the shallow area can be provided to meet the demand for fish spawning.

### 3.2. Referential Hydrologic Conditions

**Figure 6:**
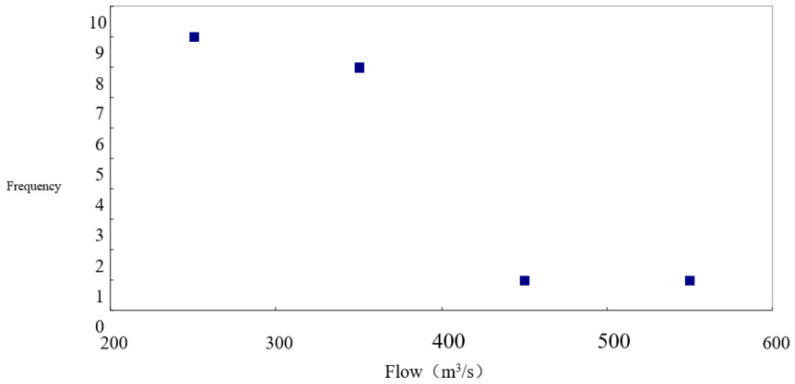
Value and occurred times at 75 percent with a frequency of 17 in the past 21 years (1948–1968). Source: China Water Resources Yearbook, 2018.

Taking 1948 to 1968 as an example, when human activities for referential hydrology series less influence the hydrologic condition, the five months (from November to March) in winter explains the flow value and occurs times with a guaranteed rate of 75% in the past 21 years (1948–1968). We can see in Figure 6 that the frequency is 17 times at most when the flow is 200–400 m^3^·s^−1^. Judging from the corresponding frequency with a guaranteed rate of 75% from November to March in the referential hydrology series, it can be concluded that the proper flow for fish from November to March is about 300 m^3^·s^−1^ in Shizuishan fracture surface. Similarly, we can obtain the required flow, frequency, and duration for the reproduction of adult fish from April to October and for the spawning of *Silurus lanzhouensis* from April to June according to referential hydrology series and different habitat conditions under different flows.

### 3.3. Calculation Results of the Ecological Water Requirement for the Fish in the Lower Reaches of the Yellow River

Based on the above calculation and analysis result, we obtain the runoff condition that meets the demand of *Silurus lanzhouensis* in different fracture surface regions, including the minimum flow and proper flow. The flow elements consist of the flow volume, duration, and frequency (see Table 2). We can obtain the ecological water volume that meets the demand for fish in the fracture surface of Shizuishan, Toudaoguai, Longmen, and Tongguan by the same research approach (see Table 2). For example, the Shizuishan region (fracture surface) is categorized into minimum and appropriate flow and then subcategorized into a low flow and pulse flow according to time distribution. The value showed that a minimum pulse flow of 400 (m^3^·s^−1^) occurs once a year and lasts for six days, while the low flow frequency results showed a continuous trend.

The above research of ecological water requirements for fish does not take its requirements for flood into account. It is essential for the flood in the living and foraging of the fish. This study considers the flood requirements for fish combined with the sediment transport in the flood season. 

## 4. Discussion

Based on the calculation and analysis above, the runoff condition met the demand of the upper and middle reaches of the Yellow River, including the minimum and proper flow. The flow elements consist of flow volume, duration, and frequency. With the same research approach, the ecological water volume met the Yellow River Dao fish migration demand. When managing the ecosystem in the lower reaches of the Yellow River, the intervention and the adjustment of the discharge model must be implemented according to the needs of the different plants at the different life stages. During the seed germination and dispersal, the necessary pulse flows must be formed to meet the needs for plant propagation. The plants in the riparian zone of the lower Yellow River can provide the habitat and food for the benthonic animals and fish in the lower reaches of the Yellow River. Therefore, only after the significant restoration of plants in the riparian zone, the benthonic animals and fish can be effectively restored.

Most of the ecological objectives in the water ecosystem from the mainstream of the Yellow River are implemented in the watercourse of the Yellow River. The ecological water requirements of a particular objective can meet the ecological water requirements of other objectives [41,42]. For instance, the water requirements of sand flushing can meet the water requirements of the offshore area and wetlands near the estuary. In other words, some ecological water requirements overlap, and some requirements can be used for many other water ecosystems in the meanwhile. Due to the calculation on the ecological water requirements of the main Yellow River stream, different magnitudes of water volume requirements that met multiple functional needs should be coupled.

### 4.1. Thought on Ecological Water Requirement Coupling

The core idea of ecological water requirements coupling is to obtain the flow duration curve to meet the multiple functional needs of the ecological water requirements [43,44,45]. The principles of ecological water requirements coupling are ① the general consideration of the whole stream segment: carry out the integration of important fracture surface flow; it should take such factors as the matching and flow routing of flows between high and low fracture surfaces and then make the coupling after comprehensive optimization; ② the consideration of water in taking and water loss: there are lots of water intakes at the mainstream of the Yellow River and their distribution is complicated. The water loss should be taken into consideration due to water evaporation and water leakage in water in taking; ③ in precedence to the assurance of water quality: it is the primary objective of river ecosystem recovery to improve the water quality. Only good quality water resources can implement other ecological functions and economic functions of the river. The demand for water quality: the definition of suitable water volume is according to the eco-environmental water volume in the viable pollution level, and the minimum water volume is taking into account the eco-environmental water volume under the target control.

Given the matching and the flow propagation between different fracture surfaces, the ecological water requirements coupling is carried out from bottom to top. That is to say, the coupling is from the Lijin fracture surface firstly, then gradually reaches the upstream fracture surface. This ecological scheduling regulation cascade is based on the Long-Liu reservoir group in the upper reaches and the San-Xiao reservoirs group in the middle and lower reaches. Later, the ecological coupling is divided into two segments, the stream segment in the lower reach of Huayuankou and the stream segment of Tongguan. The propagation time of the adjacent fracture surface can be neglected because the scale of reservoir regulation is a monthly scale. The scheduling model will consider the matching and flow propagation time of ecological flows between different fracture surfaces. In terms of optimization, scheduling is a significant part of workflow management, but other functions must be added to the scheduling system to fulfill the requirements of workflow management.

(1) $$Q_{t}={\alpha I}_{t-1}+\left(1-\alpha \right)I_{t},\;\alpha =\frac{\tau }{\Delta t}$$

In the above formula: I, Q, refers to the flow (m^3^·s^−1^) of the top fracture surface and bottom fracture surface, respectively; α is the vertical distribution coefficient of flow speed; Δt refers to the scheduling time frame; τ refers to the flow propagation time of stream segment.

In consideration of interval inflow QR and planning water QY, the balanced equation on water volume is as follows:

(2) $$Q_{t}={\alpha I}_{t-1}+\left(1-\alpha \right)I_{t}+\mathrm{QR}-\mathrm{QY}$$

Formula (2) is the balanced equation on the water volume of the fracture surface. The control and surveillance of flow propagation are based on the water volume of the fracture surface. However, the water volume of the fracture surface not only includes the ecological water requirements but also consists of the usage of water for irrigation, electricity generation, industry usage, and daily life. Therefore, the required flow duration curve of ecological scheduling is a coupling process combining the ecological water, industrial water with domestic water, and ecological flow with the economic water. The time continuity will be solved by building the ecology scheduling model of the reservoir and its solution procedure. In this part, the ecological flow process is obtained by the upper envelope of minimum and maximum values, which were regarded as the initial value of the scheduling model.

### 4.2. Ecological Water Requirement Coupling of Each Fracture Surface from the Mainstream of the Yellow River

#### 4.2.1. Other Ecological Water Requirements

Given the limited research time, the ecological water requirements used for sediment transport and self-purification adopts the existing result achieved by the Eleventh Five-Year Supporting Plan “Key technology on the restoration of Yellow River problems” and modern innovation projects from the Ministry of Water Resources. The appropriate flow value and minimum flow value of self-purification on the Yellow River’s mainstream are seen in Table 3. The ecological water requirements in the coastal wetland of the Yellow River Delta are targeted as the scale of reed wetland in 1992. The appropriate rate of water make-up is 350 million m^3^, in which the rate of water make-up in spring and summer respectively account for 1/3 and 2/3.

According to the existing results, the sediment of the Yellow River is 800 million tons, and its water volume for sediment transport is 21 billion m^3^. During the calculation of the ecological scheduling plan of the reservoir, the minimum water volume was regarded as the water volume for the sediment transport. In consideration of the flood from July to October and ensuring the sediment of 800 million tons in the future at the downstream of the Yellow River, it needs to keep the minimum water volume for a particular flow capacity of the main channel, under which the reservoir can regulate the flow and sediment entirely.

#### 4.2.2. Ecological Water Requirement Coupling Result of Each Fracture Surface from the Mainstream of the Yellow River

According to the thought mentioned above on ecological water requirement coupling, we obtain the ecological water requirement coupling result of each fracture surface from the mainstream of the Yellow River as Table 4. For example, in the Shizuishan region (fracture surface), the flow demand for fish from November to March is 150 (m^3^·s^−1^), while the demanded value for the maintenance of the eco-hydrological connection among different sections is 325 (m^3^·s^−1^) in the same duration. Moreover, required flood values are given with proper time and duration according to the fracture surfaces. 

### 4.3. Comparison of the Ecological Requirements of Water 

The definition of ecological water demand has still not been unified at present. Covich et al. [46] considered that ecological water demand referred to the water amount which was required to ensure the recovery and maintenance of healthy development of the ecological system in water resources management. Gleick [47] considered that the ecological water demand referred to providing a certain quality and quantity of water amount to the natural habitat, minimizing the changing process of the natural ecological system, and ensuring the diversity of species and the ecological integrity [23]. There are many calculation methods regarding the ecological flow. Australia, South Africa, the USA, Canada, and other forty-plus countries have already established a significant number of ecological water demand calculation methods, such as the hydrologic data-based Tennant method [23], BBM method with the comprehensive consideration of water quality and quantity [48], IFIM in combination with the hydrologic, and biological model [49].

Overseas studies of the ecological water demand have focused on the natural river ecological system or the river ecological system with a lower artificialization degree. Generally, the stabilization mechanism of the ecosystem under historical and natural conditions will be used as the benchmark of the research on ecological water demand [24]. This idea is based on the assumption that the ecological system has already adapted to natural hydrologic regimes, and the maintenance of natural hydrologic regimes effectively ensures that no regime shift will occur to the system. By contrast, the total number of China’s reservoirs ranks at the top in the world. With the gradual increase of China’s hydraulic engineering construction scale, river connectivity changes. The changing process of the hydrodynamic force (water amount) has special extension and accumulation effects upon the ecological system. Therefore, it is urgent to study the impact of hydrodynamic force (water amount) on the ecological system [50]. Hydraulic engineering will inevitably change the natural hydrologic regime. The regime shift will occur to the natural system via its natural succession and the survival of the fittest, which change the ecological water demand significantly.

Generally, European and American studies on the ecological water demand have mainly focused on the ecological water demand of rivers and were more related to the species and physical morphology of river channels. These studies of the ecological water demand of the rivers have already been relatively mature [44].

The studies on China’s ecological water demand also start from the ecological system of the river. China has started studying and exploring the river’s minimum flow problem since the 1970s, mainly focusing on the study of determination methods of the river’s minimum flow [51]. The concept of ecological water usage was first proposed at the end of the 1980s upon analyzing the water resources and Oasis construction issue of Tarim Basin in the Xinjiang Uygur Autonomous Region. In the 1990s, the concept of ecological water demand began to be recognized by China’s scientific community and government administration departments. The studies on the ecological water demand began to become domestic research hotspots. From the perspective of water resources development and utilization and the ecological and environmental mutual coordinated development, China proposed four major balance principles to calculate the ecological water demand. The National Key Technology Research and Development Program of China during the “19th Five-Year Plan”, Reasonable Utilization of Water Resources and Ecological Environmental Protection in Northwestern China, made the systematic research on the ecological water demand in dry regions and firstly proposed the calculation methods of ecological water demand researches in light of the features of dry regions [52]. Afterward, China started researching the river’s ecological water demand in the Huang-Huai-Hai Plain Region [25] based on the remote sensing and geographic information system technology and the researches of regional ecological water demand in combination with the water resources calculation theories and vegetation ecological theories. At the beginning of the 21st century, China established its regional ecological water demand theory and calculation method system and constructed the ecological water use standard technical analysis system. The ecological water usage analysis mechanism was adopted to conform to China’s special river conditions, and the critical technology for the series of regional ecological water demand calculations was developed [24].

In China, ecological water demand theories were gradually improved by various researchers. They believed that the ecological water demand of the river system should ensure that the aquatic ecosystem was under a healthy status. They proposed that the river’s ecological demand had the features of tolerance theory, which had the tolerance limit, and also had three base points, namely, the minimum, fittest, and maximum ecological water demand [27,51].

Wang Xiqin et al. [33] explored the connotation of the ecological water demand of river channels and proposed that the minimum ecological water demand referred to the perennially flowing minimum water amount threshold that maintains the river’s essential environmental functions. Wang et al. [27] thought that the exploitation and utilization of water resources should be mutually coordinated and developed with the ecological environment. He proposed that the calculation of ecological water demand should follow four major balance principles, namely, water and heat (energy) balance, water and salt balance, water and sand balance, and regional water amount supply and demand balance. Song et al. [51] considered that the ecological water demand referred to the water amount which was required for the ecological system to reach a certain ecological level or maintain a particular sort of ecological system balance, or the water amount required for realizing the expected ecological function; besides, the water amount allocation should be reasonable and sustainable; for a special ecological system, its ecological water demand had a threshold range with the upper limit and lower limit. If either the upper limit or the lower limit was exceeded, the degradation and destruction of the ecological system would occur. They considered that the ecological water demand referred to the minimum water resources required to maintain the basic survival of biocenosis of the ecological system and carry out a specific ecological construction [27,40].

Although China’s ecological water demand study is a late starter, it has rapidly developed [52]; with continuous in-depth research of China’s ecological water demand, the relevant theories gradually become mature. The ecological water demand has become a significant topic that must be considered for the environmental impact analysis during the water resources planning and allocation and hydrologic engineering construction process and has also become a critical component of aquatic ecosystem recovery and wetlands and biodiversity conservation.

## 5. Conclusions

Based on the relevant materials on the protection of ecological fish habits in the mainstream of the Yellow River, this paper studied the ecological water requirements by the flow restoration method. Specifically, it consisted of applying the River2D model (two-dimensional model of river hydrodynamics and fish habitat) and a one-dimensional model of hydrodynamics HEC-RAS to obtain the hydraulic parameters on the protection of Yellow River fish in different life stages. The objective of this paper was to analyze the relationship between the different flows and hydraulic parameters. It defines the ecological water requirements of fish in the upper and middle reaches of the Yellow River regarding the runoff condition. Ecological water requirements not only include the different flow components but also the duration, the frequency, and the distribution time. The research results are close to reality and have preferable operability. In this paper, the ecological water requirements coupling method of the main fracture surface from the mainstream of the Yellow River is put forward, and the ecological flow process is achieved by the upper envelope of minimum and maximum value that is regarded as the initial value of the scheduling model.

## 6. Limitations

Overall, it is expected that this study has some limitation which provides opportunities for further research. Remarkably, research has been put forward by considering the two-dimensional and one-dimensional model to analyze the different flows and hydraulic parameters on the protection of Yellow River fish in different life stages. We did not claim the variables used in the models were perfect in all senses; we relied on the secondary data. Moreover, the models and the nature of the data do not allow us to include other variables. Further, we only focused on the Yellow river; thus, this study recommends conducting further research in other rivers to fulfill the knowledge gap and formulate applicable profound policies.

## Figures and Tables

**Table 1:** Flow components and hydraulic indicators related with ecological demands of *Silurus lanzhouensis*.

Ecological Object	Flow Components	Hydraulic Indexes	Time Distribution
Keep a habitat at sufficient depth to help the adult fish live through the winter	Low flow	Max D > 1.2 m V: 0.4–1.6 m·s^−1^	November to March, April to June
Spawning	Low flow	D: <1.2 m shallow water area V: 0.4–0.6 m·s^−1^	From April to June

**Table 2:** Ecological flow demand of fish at the stations in Shizuishan, Toudaoguai, etc.

Fracture Surfaces	Category	Flow Components	Distribution Time	Flow (m^3^/s)	Frequency	Duration
Shizuishan	Minimum	Low flow	November to March	150	Continuous	
		Pulse flow	April to June	400	One time in a year, at least	More than six days
		Low flow	April to October	150	Continuous	
	Appropriate	Low flow	November to March	300	Continuous	
		Pulse flow	April to June	900	One time in a year, at least	More than six days
		Low flow	April to October	250	Continuous	
Toudaoguai	Minimum	Low flow	November to March	125	Continuous	
		Pulse flow	April to June	400	One time in a year, at least	More than eight days
		Low pulse	April to October	125	Continuous	
	Appropriate	Low pulse	November to March	250	Continuous	
		Pulse flow	April to June	800	One time in a year, at least	More than eight days
		Low flow	April to October	250	Continuous	
Longmen	Minimum	Low flow	November to March	130	Continuous	
		Low flow	April to October	130	Continuous	
	Appropriate	Low flow	November to March	240	Continuous	
		Low flow	April to October	240	Continuous	
Tongguan	Minimum	Low flow	November to March	150	Continuous	
		Low flow	April to October	150	Continuous	
	appropriate	Low flow	November to March	240	Continuous	
		Low flow	April to October	240	Continuous	

**Table 3:** Self-purification water requirements on the Yellow River typical cross-section at the present stage unit: m^3^·s^−1^.

Category	Time Interval	Lanzhou	Xiaheyan	Shizuishan	Toudaoguai	Longmen	Tongguan	Xiaolangdi	Huayuankou
Appropriate value	November to February	350	340	330	120	240	300	300	320
	March to June	350	340	330	120	240	300	300	320
	July to October	350	340	330	300	400	500	300	320
Minimum value	All year round	355	340	325	75	130	150	160	170

**Table 4:** Ecological water demand coupled results in the maintenance of the eco-hydrological connection among different sections (Shizuishan, Toudaoguai, etc.) on the Yellow River.

Fracture Surfaces	Category	Flow Components	Distribution Time	Flow (m^3^·s^−1^)	Frequency	Duration
Shizuishan	Minimum	Low flow	November to March	325	Continuous	
		Pulse flow	April to June	400	One time in a year at least	More than 6 days
		Low flow	April to October	325	Continuous	
		Flood	July to October	Flood 1000 ~1500		About 20 days
	Appropriate	Low flow	November to March	330	Continuous	
		Pulse flow	April to June	900	One time in a year at least	More than 6 days
		Low flow	April to October	330	Continuous	
Toudaoguai	Minimum	Low flow	November to March	125	Continuous	
		Pulse flow	April to June	400	One time in a year at least	More than 8 days
		Low flow	April to October	125	Continuous	
		Flood	July to October	Flood 1000 ~1500		About 20 days
	Appropriate	Low flow	November to March	250	Continuous	
		Pulse flow	April to June	800	One time in a year at least	More than 8 days
		Low flow	April to June	250	Continuous	
		Low flow	July to October	300	Continuous	
Longmen	Minimum	Low flow	November to March	130	Continuous	
		Low flow	April to October	130	Continuous	
	Appropriate	Low flow	November to March	240	Continuous	
		Low flow	April to October	240	Continuous	
Tongguan	Minimum	Low flow	November to March	150	Continuous	
		Low flow	April to October	150	Continuous	
	Appropriate	Low flow	November to March	300	Continuous	
		Low flow	April to October	300	Continuous	
Huayuankou	Minimum	Low flow	November to March	200	Continuous	
		Pulse flow	April to June	1400	One time in a year at least	More than 6 days
		Low flow	April to October	200	Continuous	
		Flood	July to October	3500		About 40 days
				3500 or one floods in five years 5150 and 3550		About 9 days
	Appropriate	Low flow	November to March	400	Continuous	About 28 days
		Pulse flow	April to June	1700	One time in a year at least	More than 6 days
		Low flow	April to October	800	Continuous	
Lijin	Minimum	Low flow	November to April	70~80	Continuous	
		Pulse flow	April	400	Continuous	
		Low flow	June to October	200~300	Continuous	
		Flood	July to October	3500 or one floods in five years 5150 and 3550		About 40 days About 9 days About 28 days, Respectively
	Appropriate	Low flow	November to March	120~290	Continuous	
		Pulse flow	April	1800	Continuous	
		Low flow	June to October	500~700	Continuous	

## Data Availability

Data available in a publicly accessible repository.
